# What do patients with diabetes and providers think of an innovative Australian model of remote diabetic retinopathy screening? A qualitative study

**DOI:** 10.1186/s12913-017-2045-2

**Published:** 2017-02-22

**Authors:** Nicola M. Glasson, Sarah L. Larkins, Lisa J. Crossland

**Affiliations:** 1 College of Medicine and Dentistry, James Cook University, 1 James Cook Drive, Townsville City, QLD 4811 Australia; 2 Discipline of General Practice, University of Queensland, Royal Brisbane Hospital, Level 8 Health Sciences Building, Herston, QLD 4029 Australia

**Keywords:** Diabetic retinopathy, Screening, Rural and remote, Qualitative study

## Abstract

**Background:**

Diabetic retinopathy (DR) is the commonest cause of preventable blindness in working age populations, but up to 98% of visual loss secondary to DR can be prevented with early detection and treatment. In 2012, an innovative outreach DR screening model was implemented in remote communities in a state of Australia. The aim of this study was to explore the acceptability of this unique DR screening model to patients, health professionals and other key stakeholders.

**Methods:**

This descriptive qualitative study used semi-structured interviews with patients opportunistically recruited whilst attending DR screening, and purposefully selected health care professionals either working within or impacted by the programme. Interviews were audiotaped, transcribed and analysed using NVIVO. An iterative process of thematic analysis was used following the principles of grounded theory.

**Results:**

Interviews were conducted with fourteen patients with diabetes living in three remote communities and nine health professionals or key stakeholders. Nine key themes emerged during interviews with health professionals, key stakeholders and patients: i) improved patient access to DR screening; ii) efficiency, financial implications and sustainability; iii) quality and safety; iv) multi-disciplinary diabetes care; v) training and education; vi) operational elements of service delivery; vii) communication, information sharing and linkages; viii) coordination and integration of the service and ix) suggested improvements to service delivery.

**Conclusions:**

The Remote Outreach DR Screening Service is highly acceptable to patients and health professionals. Challenges have primarily been encountered in communication and coordination of the service and further development in these areas could improve the programme’s impact and sustainability in remote communities. The service is applicable to other remote communities nationally and potentially internationally.

**Electronic supplementary material:**

The online version of this article (doi:10.1186/s12913-017-2045-2) contains supplementary material, which is available to authorized users.

## Background

Diabetic retinopathy (DR) is the commonest cause of preventable blindness in working age populations, with close to 30% of persons with diabetes over 40 years of age impacted [1, 2]. Indigenous^1^ peoples are particularly at risk, with the incidence of blindness six times higher in Indigenous than non-Indigenous Australians [3, 4]. This is despite evidence that early detection and timely treatment, can prevent up to 98% of visual loss secondary to DR [5]. Documented DR screening rates are disappointing internationally, with few countries reporting effective screening programs [6, 7]. Less than 50% of Australian and American patients with diabetes receive appropriate screening, with rural and remote communities with poor access to ophthalmology services particularly at risk [6–9]. As the number of people with diabetes continues to increase relentlessly worldwide, lack of effective DR screening poses a significant public health and economic challenge [1].

**Fig. 1:**
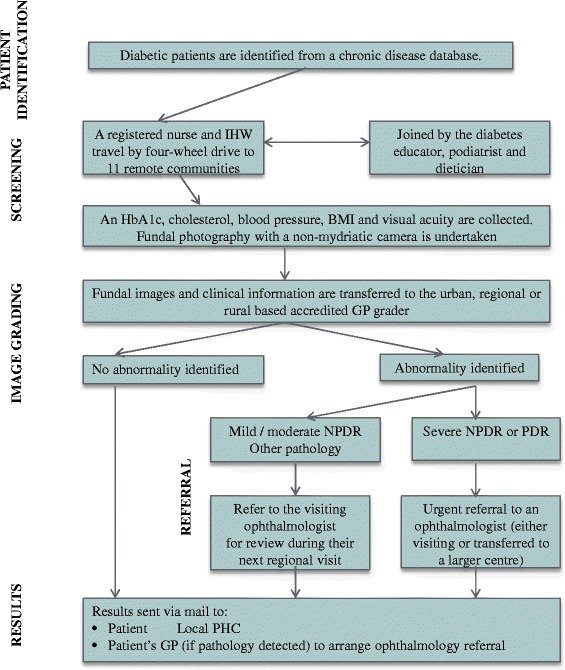
The Remote Outreach DR Screening pathway. *Adapted from Glasson *et al. [11]. *Note: IHW (Indigenous Health Worker), BMI (body mass index), PHC (primary health care centre), NPDR (non-proliferative DR), PDR (proliferative DR), GP (general practitioner)*

The Remote Outreach DR Screening (RODRS) Service was implemented in 2012 in remote Australia (Fig. 1). DR screening with a non-mydriatic retinal camera was conducted by a multi-disciplinary diabetes service already visiting eleven remote communities. Remote communities are located between 117 km and 693 km (approximately 1.5 to 7 h drive) from the rural hub, which itself is located 1176 km from the state capital [10]. Communities have a documented diabetic population between 3 and 49 persons. A distant general practitioner (GP) reviews and grades images and suggests appropriate management. An urban-based ‘buddy’ ophthalmologist supports the GP grader and provides visiting services to the region, following up screen-positive patients. In 2014, a retrospective, descriptive study was undertaken in order to: i) identify the percentage of patients with diabetes mellitus (type 1 or type 2) who received appropriate retinal screening prior to and following the introduction of the RODRS programme; ii) identify the proportion of patients with mild, moderate or severe non-proliferative DR (NPDR) and proliferative DR (PDR); and iii) explore the acceptability of the programme to patients and health professionals [11]. A detailed description of the model and results of the quantitative study have been outlined in a separate publication [11].

A systematic review by the authors identified four components common to effective rural remote DR screening models, namely: accessibility; integration of the service with the broader health system; communication and coordination of care; and patient acceptability [12]. This paper presents results from the qualitative section of the study, focusing on the acceptability of the outreach screening programme to patients, health care providers and other stakeholders and investigates the extent to which the model aligns with components common to effective rural remote DR screening models [12].

## Methods

This descriptive qualitative study was conducted between February 2015 and July 2015 as part of a broader programme evaluation. It was approved by Queensland Health Ethics (HREC/15/QRBW/125). The acceptability of the RODRS service was explored using in-depth, semi-structured interviews with patients, local and regional health providers involved in or impacted by the RODRS service. All patient interviews were conducted onsite face-to-face in clinics, during screening visits. Health provider and key stakeholder interviews were conducted through a mixture of onsite face-to-face and telephone contact. The patient interviews explored patient understanding of DR and the RODRS programme, acceptability of the screening programme and areas for improvement. Interviews with health professionals explored their understanding of the service, their experiences working with this model of care, perceived benefits of the programme and areas for improvement (Additional file 1). The interviewer was not involved in the implementation of the RODRS service.

### Patient, health professional and stakeholder recruitment

Patients were opportunistically recruited whilst attending DR screening with the RODRS service. Patients were invited to participate if they were 18 years of age or older, with type 1 or 2 diabetes mellitus, attending DR screening and with sufficient cognition to provide informed consent. Patients were excluded from participation if they had no perception of light in either eye; were too unwell to participate; or had a physical or mental disability that prevented either screening or treatment. Health professionals and other key stakeholders were identified using a purposeful sampling strategy to provide a rich overview of the RODRS programme. The study was explained to interview participants and written informed consent was obtained prior to data collection. No patients or health professionals refused to participate.

All interviews (face-to-face and telephone) were audiotaped and transcribed. Additional observational data on the process of screening (including patient appointment and attendance processes) was recorded by the researcher during on-site visits.

### Analysis

All interviews were transcribed and coded by one member of the research team (NG) using NVivo software (version 10.2) [13]. Codes were reviewed by a second member of the research team (LC) for duplication and clarity. An iterative process of thematic analysis was used to identify and classify recurrent patterns and themes [13].

## Results

This section provides an overview of the interview participants and presents recurrent themes identified during semi-structured interviews.

### Interview participants

#### Health professionals and key stakeholders

Six health professionals participating in the screening programme and three key stakeholders anticipated to be impacted by the implementation of the service were interviewed (Table 1). Health professionals included registered nurses involved in programme coordination and DR screening, a diabetes educator, an Indigenous outreach worker, a GP grader and the ‘buddy’ ophthalmologist. Key stakeholders interviewed included a regional visiting optometrist and ophthalmologist and the director of the health district.

**Table 1:** Health professionals and key stakeholder interview participants

Position	Role in the programme
*Health professionals*	
Eye screening coordinator	Coordinates the screening programme from the rural hub and visits remote communities to capture fundal images
Registered nurse screener	Visits remote communities to capture fundal images
Diabetes educator	Travels with the DR screening team providing diabetes education
Indigenous outreach worker	Contacts local people with diabetes in remote communities and is involved in screening visits
General practitioner grader	Receives fundal images, identifying and grading DR and suggesting appropriate management
‘Buddy’ ophthalmologist	Provides support to the programme and follows-up screen-positive patients during visits to the region
*Key stakeholders*	
Visiting ophthalmologist	Provides visiting ophthalmology services to the region
Optometrist	Provides visiting optometry services to three remote communities visited by the RODRS programme
Director of the health district	Leader of health service coordination in the region

#### Patients

Semi-structured interviews were conducted with 14 patients in three remote communities serviced by the programme (Table 2). A total of 64% of participants were female. The majority of participants were Indigenous (71%), which was higher than the total Indigenous population screened by the service (25%). This was due to the fact that interviews were conducted in communities with high Indigenous populations, but this group was also purposefully targeted due to lower DR screening rates. Patients ranged from 20 to 81 years of age. Duration of diabetes ranged from less than one year to 25 years. Most participants had previously undergone DR screening (79%) and 64% of participants had been screened by the RODRS service previously.

**Table 2:** Patient interview participants

	Age	Gender	Ethnicity	Duration of diabetes (years)	Previous DR screening with the service	Pathology detected	Previous DR screening with other providers
1	20	Female	Aboriginal	<1	No	-	No
2	44	Female	Aboriginal	1	No	-	Ophthalmologist
3	42	Female	Aboriginal	6.5	No	-	Ophthalmologist
4	81	Male	Non-Indigenous	>20	No	-	Unclear
5	35	Male	Aboriginal	4	Yes	No	Ophthalmologist
6	38	Male	Aboriginal	7	Yes	Moderate NPDR	Ophthalmologist
7	68	Female	Non-Indigenous	<1	No	-	No
8	31	Female	Aboriginal	6	Yes	No	Ophthalmologist & optometrist
9	67	Male	Non-Indigenous	4	Yes	Inadequate image	Ophthalmologist
10	53	Male	ATSI	4	Yes	No	Ophthalmologist & optometrist
11	56	Female	Aboriginal	>10	Yes	Severe NPDR	No
12	52	Female	Non-Indigenous	4	Yes	Mild NPDR	Ophthalmologist
13	49	Female	Aboriginal	25	Yes	Mild NPDR	Ophthalmologist
14	50	Female	Aboriginal	>5	Yes	Inadequate image	Ophthalmologist & optometrist

*Note: ATSI (Aboriginal and Torres Strait Islander), NPDR (non-proliferative DR)*

### Themes

**Fig. 2:**
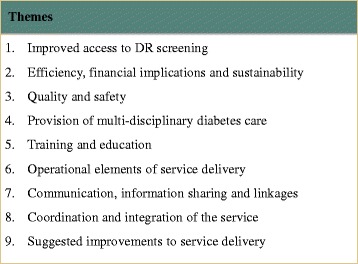
Themes identified from patient, health professional and key stakeholder interviews

**Fig. 3:**
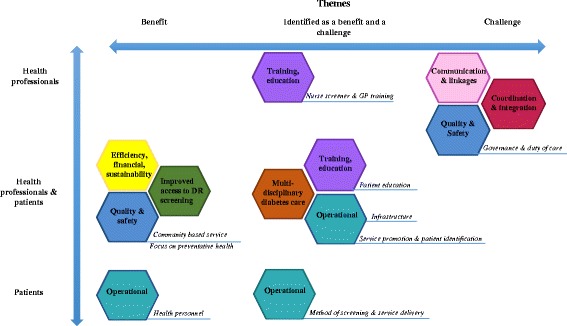
A visual representation of themes identified from patient and health professional interviews grouped into benefits and challenges of the RODRS service. *Note: Theme 9 (suggested improvements to service delivery) is not represented. Note: The term health professional incorporates themes identified in both health professional and key stakeholder interviews*

Nine key themes emerged during interviews with health professionals, key stakeholders and patients (Fig. 2). These represented a combination of benefits, challenges and suggested improvements to service delivery. Figure 3 presents an overview of the interrelation of themes and whether these were perceived as benefits or challenges by health professionals^2^ and patients. All themes that emerged during patient interviews were identified as themes or subthemes in health professional interviews, except for those that related to the operation of the programme (Theme 6).

### Theme 1: improved patient access to DR screening

Both health professionals and patients recognised improved accessibility to DR screening as a key benefit of the programme. Health professionals acknowledged that without the visiting screening service, many patients with diabetes would not undergo screening due to large travel distances, lack of transportation and negative financial consequences. Patients mentioned ease of access in terms of the close proximity of the service to their homes and avoidance of the need to travel. This was identified as particularly important due to large travel distances, a lack of public transport, no access to a car and for those who were disadvantaged or unwell. In addition, interviewees mentioned the direct transport of patients from their homes to the screening clinic as a positive aspect of service delivery. Improved access was identified as important for the working population who previously had difficulty accessing out-of-town screening services due to their operation during working hours.

*A lot of these people in these communities just don’t have the means and probably wouldn’t drive to a bigger centre to have their eyes checked… if we didn’t provide this service there would be a large percentage of these people who just would never get reviewed. [Health professional]*

*Not everyone has a car to get away or can afford to get away. [Patient]*

### Theme 2: efficiency, financial implications and sustainability

Both health professionals and patients recognised greater efficiency and potential positive financial implications as key strengths of the programme. Health professionals recognised the model as efficient and economical, utilising the scarce specialist workforce more effectively for treatment rather than screening, saving on the patient travel subsidy scheme (PTS) and delivering a preventative health service that saves the health dollar in the future. Patients identified beneficial financial implications including reduced travel costs and avoidance of lost income associated with absences from employment.

*I definitely think it is economical - you are saving on PTS, by doing the screening you are picking up problems, you can intervene and that is saving the healthcare dollar down the track. [Health professional]*

*It’s a rational use of resources and it should be a more cost effective. [Health professional]*

*You have got to take a day off work, that’s lose a day’s pay, plus expenses going up. So sometimes it is just beyond your pocket. [Patient]*

All health professionals perceived the model as sustainable and mentioned it could be successfully trialled in other rural and remote communities. One health professional acknowledged that the model needed to be flexible and adapt to local needs and integrate with other health services to continue to be locally appropriate. All patients found the model acceptable and intended to return to the screening service, except for one interviewee who was dissatisfied with the ‘flash of the camera.’

*I think it is a great model and it should be emulated elsewhere around Australia. [Health professional]*

*I think it is a sustainable model… some flexibility in the ability of the service to grow closer to what’s been delivered locally… one of the problems you have particularly with any federally delivered service…they have such rigid rules around delivery of service that they run that they actually become counterintuitive, counterproductive and ineffective at the local level because they don’t allow the local tweaking that is necessary. [Health professional]*

### Theme 3: quality and safety

Both health professionals and patients identified community-based service delivery and a focus on preventative health as two key benefits of the DR screening programme. Health professionals recognised appropriate clinical governance and duty of care as a challenge of the service.

The RODRS service was recognised by health professionals and patients as a community-based and locally appropriate screening service. Health professionals mentioned the use of local health practitioners led to community ownership of the programme. Patients perceived the programme as equitable, delivering screening to both Indigenous and non-Indigenous residents, which met community needs.

*This new model allows ownership of the system, involves the people… the GPs have ownership of the programme and the nurses have ownership of the programme and therefore they are more likely to want to sit down and make it work…its about devolving responsibility back to the local level. [Health professional]*

*I have seen people coming up here to this test who would normally never come and it is all because of the project delivering to the community needs. [Patient]*

Both health professionals and patients identified the delivery of a preventative health service to be a major benefit of the model. Benefits mentioned by health professionals included prevention of diabetic-related blindness, detection of other complications of diabetes, detection of other cardiovascular risk factors (such as hypertension and dyslipidaemia) and detection of other ophthalmic pathology. Patients specifically mentioned prevention of diabetic-related blindness to be a benefit of the service.

*People out here tend to present late with things… often it is too late to treat. So certainly treating things before they become symptomatic and irreversible is a positive. [Health professional]*

*I have had to go away now and have laser treatment done, I think it was good that it was picked up early…I could have had more problems. [Patient]*

The duty of care of the GP grader and clinical governance were identified as challenges of the service. Responsibility for patient follow-up was mentioned as an area of uncertainty and was contributed to by suboptimal communication and internal linkages within the model, particularly with local GPs.

*One of the issues that I slightly had was a bit of governance…when [the patient’s] measurements are clearly out of normal range and they really need a general practice follow-up, there is not any way of referring them to a GP … if the information is clear that the patient shouldn’t be driving, but I don’t know because they are not in front of me…I wonder about the duty of care. [Health professional]*

### Theme 4: provision of multi-disciplinary diabetes care

The delivery of a comprehensive, multi-disciplinary diabetes service was recognised by health professionals as a key contributor to the success of the model; however, some health professionals raised concerns with the service. Identified benefits included reduced visits to the health centre and an associated decrease in patient travel, inter-disciplinary education of staff and improved team morale. In addition, health professionals commented that high attendance rates for retinal screening enabled allied health practitioners to see patients who were otherwise non-compliant with appointments.

*It is a one-stop shop. It is much more attractive if you are travelling significant distances to be seen by the team and also the importance of that is that we all learn from each. [Health professional]*

*A lot of these people live in remote locations…so we try to make it easy for them to achieve good healthcare in that they only have to come into the clinic one day…people that wouldn’t normally come in to see a dietician or a podiatrist, because they are there on the day getting their images reviewed, they will go in and have a foot check and have a discussion about their diet. [Health professional]*

However, some health professionals reported that the need to see multiple health practitioners led to time inefficiencies, reduced capacity for retinal screening and was logistically challenging given limited space in small clinics. Some health practitioners believed it was time consuming and intrusive for patients and that patients were not retaining the large volume of advice given to them. One suggested improvement was to travel with the diabetes educator only who would perform a simple foot examination and diet review, referring high-risk patients to allied health.

*I sometimes feel that we are doing this mass screening and we are rushing patients through…how much of the education with the dietician do they actually take home…and that’s not a good experience for the patient. [Health professional]*

*Whether we do a simple foot check and have a discussion on diet and refer anyone who is high risk. [Health professional]*

In contrast, some health professionals reported that large multi-disciplinary clinics provided a community gathering point and an opportunity for social interaction. They recognised the extended clinic times as a benefit given that some patients were undergoing pupil dilation, with most needing to drive following their appointment. Whilst they commented that the logistics were challenging, they believed that this was overcome by staff flexibility and utilisation of outdoor spaces. They also reported that given it was promoted as a ‘super-clinic’ with multiple health professionals, local employment agencies were more likely to give patients time off work. This was supported by patient interviews, which did not identify negative experiences with a multi-disciplinary model of care. One patient considered it a key benefit of the programme.

*They were warned that it was probably going to take a couple of hours…most people found it actually quite social… sometimes it was a little crowded in the clinics but…people were happy to sit out and have a cuppa.…and people were pretty happy that they had attended the ‘super-clinic’ and had their feet and eyes… their eyes were probably going to be dilated…most people had to drive afterwards so that was a bit of a bonus to have them hanging around for a while. [Health professional]*

### Theme 5: training and education

The need for comprehensive and continued training of registered nurse screeners and GP graders were key themes that emerged during interviews with health professionals. In addition, health professionals and patients identified patient education to be a key benefit and further challenge of service delivery.

The lack of formal and ongoing training of nurse screeners was identified as a key challenge. Whilst interviewees reported that the automated camera was easy to operate, they stated there was insufficient tuition provided on image adequacy and the characteristics of a pathological image. They commented that better training could increase image quality and improve patient follow-up by allowing screeners to flag patients with obviously abnormal images, emphasising the importance of receiving timely results to local staff and patients. Interviewees suggested a process of feedback to screeners from the GP grader and/or ophthalmologist on image adequacy could assist with further refinement of screening technique.

*We had absolutely no training in what was a normal photograph and what was an abnormal photograph… at the very least take a photograph, say it is technically a good photograph and secondly it is normal or be able to indicate to the person this is an abnormal photograph … I could leave them a list and say make sure that you have had a report on this photograph for these people within 12 weeks…I think that would fix some of the problems. [Health professional]*

*It is a bit like the analogy of the radiologist and the radiographer. The radiographer if they know what the pathology is and what they are looking for they can get a much better image for the radiologist to assess. [Health professional]*

GP graders reported that formal training provided through a Masters of Medicine course was practical and of excellent quality; preparing them for detecting and grading DR. In addition, they commented that training improved their clinical practice independent of the programme and enabled them to provide informal education to GP co-workers. However, detection of other ophthalmic pathology was recognised as an ongoing challenge, with training provided informally by the ‘buddy’ ophthalmologist.

*We have a retinal camera here in the practice…I can look at a photo and say that it is an adequate photo and whether there is any significant pathology or not. [Health professional]*

*The GP grader is going to present a series of cases that she had actually screened, felt to be abnormal… she then educates the other GPs in the district. [Health professional]*

Health professionals commented that screening clinics provided an opportunity for patient education in diabetes management and general preventative health. Similarly, patients recognised improved awareness of the ophthalmic complications of diabetes as a benefit of the model. They commented that screening clinics created a focal talking point for the community, which could increase patient attendance in the future. However, one patient believed that more community awareness programs were needed to improve health literacy.

*It empowers the patient because they can actually see the images…it helps to reinforce the message to the patient about the importance of good sugar control, cholesterol, blood pressure and obviously no smoking. [Health professional]*

*It has created a focal talking point between the community … maybe ones who didn’t participate this time will next time because the words out there. [Patient]*

### Theme 6: operational elements of service delivery

The theme, operational elements of service delivery, included five subthemes. Health professionals and patients recognised service promotion and infrastructure to be both successes and challenges of the RODRS programme. In addition, patients identified the method of screening and health personnel as key benefits of the service. Health professionals reported that the implementation of the RODRS programme had not negatively impacted on their ability to carry out other clinical duties.

#### Service promotion and patient identification

Promotion of the RODRS service to patients and local health professionals and identification of diabetic residents eligible for and in need of DR screening within local communities were key operational elements that emerged during interviews. Whilst most patients were notified of screening visits by their local health service, house calls on the day of screening, community flyers, phone calls, letters and word-of-mouth were also promotion strategies mentioned by patients. Health professionals indicated that the methods of service promotion employed differed in each community due to local factors such as lack of mobile phone reception, difficulties with postal services or poorer health literacy.

*[The local health worker] came down a week before and prepared people that were coming and took names and then today the actual people doing the test came down. [Patient]*

*The clinics that are out in the communities are probably the ones that help drive it because we are just all visiting. [Health professional]*

Health professionals reported that clinics were generally well attended, with awareness of the screening service and patient attendance gradually improving since the programme’s implementation. However, interviewees commented that further promotion of the service and better clinic planning could improve screening rates. In addition, health professionals identified poor awareness of the DR screening service amongst local GPs to be a barrier to effective service delivery. This was recognised as particularly challenging given the absence of permanent local GPs and the high turnover of locum GPs visiting remote communities. One suggestion was to integrate information about the retinal screening service into the ‘MAP of medicine’ IT system,^3^ soon to be implemented across the district [14]. GPs who use a diabetes management pathway would then be informed of, and able to refer patients to the RODRS service.

*I think everyone that is happy to come up and acknowledge they have diabetes that is in town is attending… it is a service that is really well attended. [Health professional]*

*A lot of the GPs in this district don’t actually know the screening system exists… a lot of the patients in our district don’t have regular GPs; they have locums as GPs. [Health professional]*

Patients suggested possible improvements to service promotion could include phone calls, more public notices, mailed information and SMS reminders. In contrast, some of these methods were mentioned by health professionals as difficult due to poor mailing services and the lack of mobile phone reception. Health professionals suggested newspaper advertisements and local radio announcements could be used to promote the service.

*Send a flyer to the person…you can put it on your fridge… an SMS, like the day before… it just lets people know then and remind them. [Patient]*

#### Infrastructure

Health professionals identified infrastructure limitations as a barrier to effective service delivery. This included the lack of suitable rooms for imaging given the small size of PHCs, problems coordinating with existing service delivery at PHCs and issues acquiring high quality images due to difficulties darkening clinic rooms. In addition, transportation of the heavy, bulky camera was identified as a challenge. In contrast, patients reported the small and intimate clinic size and provision of air conditioning as factors contributing to the success of the screening service.

*When we go to the PHCs obviously there is limited space, they are not like big hospitals, they are only small demountable buildings half the time. [Health professional]*

#### Health personnel and service provider

Patients mentioned the friendly, caring and knowledgeable staff as a factor contributing to the success of the service. The majority of patients had no preference for which health professional performed their DR screening (a registered nurse, optometrist or ophthalmologist), preferring instead to visit the closest service at a time when they are due for retinal screening. One participant preferred to visit the ophthalmologist due to the instantaneous results and their ability to provide treatment during the same visit. Two participants preferred the RODRS programme, as it was a community driven service. Some patients stated they would attend multiple eye services if they visited their community. Health professionals reported that many patients travelled from their community to see the district’s visiting ophthalmologist due to familiarity and high levels of satisfaction with the service.

*It is asking you your opinions, educating you about things in a humane way…I feel that this project is about the care of the people. [Patient]*

*There is not really much of a difference, just as long as my eyes are checked. [Patient]*

#### Method of screening and service delivery

Patients mentioned the simplicity of service delivery in terms of ease of access, timeliness and the simplicity of the screening procedure as benefits of the programme. Two patients were dissatisfied with the screening procedure, disliking the dilating eye drops and the flash of the camera respectively. In contrast, one patient particularly enjoyed undergoing photographic screening.

*Quick and simple. [Patient]*

*I wish all of them could do away with the drops but it’s probably not possible. [Patient]*

*I like how they take photos. [Patient]*

#### Impact on health professionals

Health professionals reported that the implementation of the RODRS programme had not negatively impacted on their ability to carry out their other clinical duties. GP graders did not report an increased clinical burden due to DR image grading and commented that training had improved their knowledge and practice as a GP. The visiting ophthalmologist reported improvements in their capacity to see patients with other ophthalmic conditions. The optometrist did not report a change in patient appointments since the implementation of the service.

*I am part time in the office so it is something that I can do from home… so it hasn’t taken any clinical time that I would be at work. [GP grader]*

*[The patients] couldn’t get in to see us and therefore they were missing out being screened and so this has allowed us increased capacity because these patients are already screened out of the system and therefore there is no need for them to see us. [Ophthalmologist]*

*Pretty stable. Hasn’t really gone up or down… I don’t know how we would get anymore in, when we are in town. [Optometrist]*

### Theme 7: communication, information sharing and linkages

**Fig. 4:**
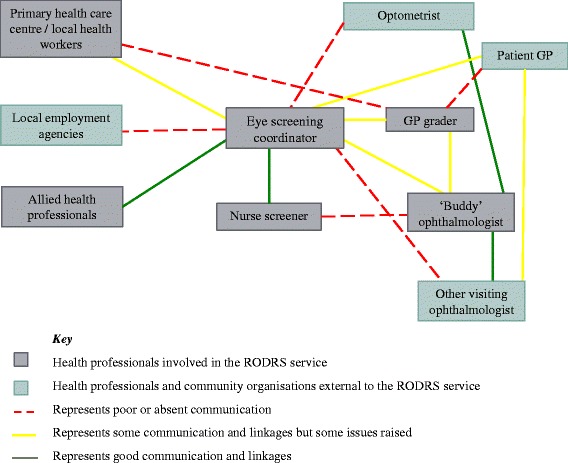
Summary of the levels of communication and linkages between health professionals and community organisations. *Note: If levels of communication and linkages were not mentioned by interviewees no line has been shown*

Communication, information sharing and linkages between health professionals working in the RODRS service and external linkages to other health practitioners and community organisations, were identified as key challenges of the programme. Figure 4 provides a summary of communication and linkages between various health professionals and community organisations.

Health professionals identified varying levels of communication and internal linkages between health practitioners working within the RODRS programme. Informal channels of communication between the eye screening coordinator, GP grader and ‘buddy’ ophthalmologist were described as a major strength of the service. However, interviewees suggested that more formal methods of regular communication were needed, such as email groups and regular video-conferencing. High staff turnover was recognised as a barrier to effective communication.

*We are a pretty good team that communicates well. [Health professional]*

*We do have a pretty open line of communication between [the GP grader] and [the eye screening coordinator]… I know [the GP grader] recently just went down and saw [the ophthalmologist] when he was visiting. So there are informal channels of communication. [Health professional]*

*It could be better. Maybe even an email group…most of it is non-urgent communication. [Health professional]*

Health professionals perceived communication with the majority of health providers and community organisations external to the RODRS programme as suboptimal.

Poor linkages were identified between the GP grader and local GPs visiting remote communities. Key contributors identified included the high turnover of locum GPs and the large proportion of patients without a regular GP. This was particularly challenging when arranging follow-up for patients with non-ophthalmic issues (e.g. hypertension), identified by the GP grader. Limited information sharing between the screening programme and local GPs was also identified as an issue, with screening results only sent to the patient’s GP if an ophthalmology referral was required. Interviewees suggested sharing the screening results of all patients to prevent double handling related to completion of the diabetes annual cycle of care, improve care and increase awareness of the programme amongst local GPs.

*When [the patients] measurements are clearly out of normal range and they really need a general practice follow-up, there is not any way of referring them to a GP… a lot of the patients in our district don’t have regular GPs, they have locum’s as GPs. [Health professional]*

*It could be simple, a template to say that your patient has had screening … it would stop double handling and it would improve the all rounded care…tick that they have had some sort of eye review in this two year cycle… it is important for chronic disease management. [Health professional]*

Poor communication and information sharing with the visiting ophthalmologist, external to the programme, was identified as an area for improvement. It was suggested that screen-positive patient lists and screening results should be distributed to both visiting ophthalmologists.

*I am not always aware that [the patients] are coming, that they have been screened… I think I could be more involved… they could email a copy of the photos. [Health professional]*

One health professional perceived communication with visiting optometry services as suboptimal. This was recognised as leading to poor integration of optometry and DR screening services, particularly in relation to the timing of screening visits, resulting in patients with diabetes not presenting to the optometrist for separate eye conditions.

*I have had it mentioned to me that the diabetics are a bit sick of coming in for repeated testing… you actually start getting attendances dropping off, that service then ceases to exist. [Health professional]*

Health professionals suggested that information sharing could be improved by use of an electronic clinical database, accessible by all health providers. This is soon to be implemented in the district. It would record the diabetic patients in each community and enable better tracking of screen-positive patient follow-up. It could potentially prevent over-servicing by ensuring patients aren’t undergoing screening with multiple service providers (RODRS service, optometry and ophthalmology). It was suggested it could also assist the GP grader by providing access to previous fundal images and ophthalmology notes, preventing unnecessary ophthalmology referrals.

*Making sure that each of those communities have their database. They should know every diabetic in their community…it is about making sure we have proper exchange of information between the parties concerned. [Health professional]*

*That [electronic database] is going to make a big difference in terms of availability of data and communication of the chronic disease management plan to all the providers. [Health professional]*

### Theme 8: coordination and integration of the service

Coordination of the RODRS programme and integration of the service with the broader health system were identified by health professionals as major challenges. This theme was fundamentally interlinked with the establishment of effective communication channels (Fig. 4). Identified challenges included providing timely follow-up of screen-positive patients, long time frames from image capture to results feedback, coordination of screening visits and fragmentation of eye care delivery in the region. A major barrier to establishing a highly coordinated service was poor workforce continuity.

Providing timely follow-up of screen-positive patients was identified as a challenge, with interviewees commenting that many screen-positive patients were not receiving an ophthalmology review. Poor workforce continuity, particularly of local PHC staff, visiting GPs and the eye screening coordinator were identified contributors. Interviewees differed in whom they perceived as responsible for coordinating follow-up of screen-positive patients (the PHC, the GP, the patient or the eye screening coordinator/nurse screener). Health professionals described difficulties expressed by PHC booking staff integrating appointments for screen-positive patients with general ophthalmology patients, due to the large number of patients requiring specialist review. Interviewees suggested that the visiting ophthalmologists should be notified of patients requiring follow-up and should then communicate their successful review back to the eye screening coordinator. Another suggestion was to use a ‘medical friend.’ This new role, soon to be implemented across the district, was described as a community member responsible for engaging patients and coordinating follow-up.

*[The patient] had letters filed in their chart saying they needed to come back for review to be seen by the ophthalmologist and no further action had been taken… whether that just sort of got missed due to there not being a person in the job. [Health professional]*

*We want to devolve a lot of those actions to what you might call a ‘medical friend.’ They are a group of people who are unskilled, actually within the community, to do the little bits that keep falling over like delivering the letters, getting people to appointments, discussing with them at the local pub why it is important for them to follow through. [Health professional]*

Poor coordination was also mentioned in the context of long timeframes from image capture to results feedback.

*There seemed to be a big gap between the time the images were taken and the reports came back. [Health professional]*

The coordination of screening visits to remote communities was also recognised as an area for improvement. High staff turnover, resulting in periods without an eye screening coordinator, was mentioned as leading to rushed planning of some visits. The timing of screening visits needed to be more cognisant of, and coordinate with local PHCs, local employment agencies, optometry visits and ophthalmology visits to remote communities. Coordination of screening clinics with ophthalmology clinics was identified as important to ensure screen-positive patients had the ability to access timely ophthalmology follow-up close to their community. There were conflicting suggestions between health providers as to the optimal timing of screening visits in relation to ophthalmology visits ranging from one month to six months.

*No one was in the job for a while, so the retinal screening kind of got left for a little bit… the planning was rushed. [Health professional]*

*When we plan our dates to go out we will need to coordinate with the times for the council work crews to make sure that anybody that is in the work group will be in town on the days that we are going. Again it is just better coordination and better communication between the service providers. [Health professional]*

Health professionals mentioned service overlap and fragmentation of eye care delivery in the region. This resulted in poor use of visiting ophthalmology services, unnecessary patient travel to access screening or, in some cases, over-servicing with patients accessing screening more than once annually through multiple service providers (visiting ophthalmologists, visiting or distant optometry services, RODRS programme). Interviewees suggested this could be prevented by improved service promotion and use of electronic databases.

*We had a lot of patients come in and they didn’t understand what the retinal screening was about until they got here… there were a few who said oh look we saw [the ophthalmologist] …they had to drive maybe 100 km to get there for their screening and the second thing is had they had their screening done with us then that frees [the ophthalmologist] up. [Health professional]*

### Theme 9: suggested improvements to service delivery

Potential improvements to the RODRS programme, as identified by health professionals, included co-service delivery and the expansion of service delivery.

#### Co-service delivery

Health professionals proposed the co-location and operation of parallel ophthalmology and DR screening clinics in communities with visiting ophthalmology services. Potential benefits included the ability to directly refer patients to an ophthalmologist when an adequate image could not be obtained, and/or if additional pathology was identified. In addition, it would facilitate communication between services, the coordination of care, support the ongoing education of screening nurses and provide a more community-orientated eye service by using local health staff. Interviewees varied significantly in regards to which health practitioners (optometry, podiatrist, dietician, endocrinologist) should be included. However, all health professionals recognised the diabetes educator as an integral part of the team. One health professional did not support a co-located service due to difficulties coordinating patient appointments and the potential that screen-positive patients (as identified by the GP grader) may have to travel to a distant centre for follow-up, given that the annual ophthalmology visit to their community had already taken place.

*My preferred model is that when we visit those centres that the diabetic educator and the clinical nurse photographer should be with us at the same time…If they need to be seen by an ophthalmologist, the ophthalmologist is there. It is a one-stop shop. [Health professional]*

#### Expansion of service delivery

Health professionals suggested the expansion of the service to screen for other pathologies, to screen for DR in larger rural townships and to increase the frequency of visits to larger remote communities currently visited by the programme. Two interviewees suggested performing retinal imaging for other pathologies, such as hypertensive retinopathy, and viewed it as an efficient use of existing resources. However, one health professional felt the programme should solely focus on diabetic eye disease. Some interviewees believed the service should be extended into larger rural communities in the district due to patient demand and to make more effective use of limited ophthalmology resources. Interviewees suggested that rural clinics could be delivered either through co-service delivery with ophthalmologists or in permanent GP practices to assist linkages with the GP grader and management of non-ophthalmic issues such as hypertension. Health professionals and patients also suggested that larger remote communities currently serviced by the programme should be visited more frequently than once annually, providing patients who are out of town or unwell at the time of the visit, another opportunity to access DR screening.

*It would decrease the number of patients [the ophthalmologist] needs to see which would be advantageous. We screen a lot of diabetics with no retinopathy… we could probably save them a trip. [Health professional]*

*If the programme gets rolled out into areas, like in the bigger towns, where there are permanent GPs, then that could be done in and amongst the GP clinic so that if there are other things picked up at the time they can be addressed. [Health professional]*

*More visits. [Patient]*

Perceptions about where the RODRS service was best targeted were varied, and included restricting the RODRS programme to those communities where there were no visiting optometry services or targeting communities without permanent optometry services.

*I think it needs to simply be restricted to areas that do not have permanent optometrists… I think retinopathy screening itself, regardless of who does it, should be available at least annually in every community. [Health professional]*

## Discussion

**Fig. 5:**
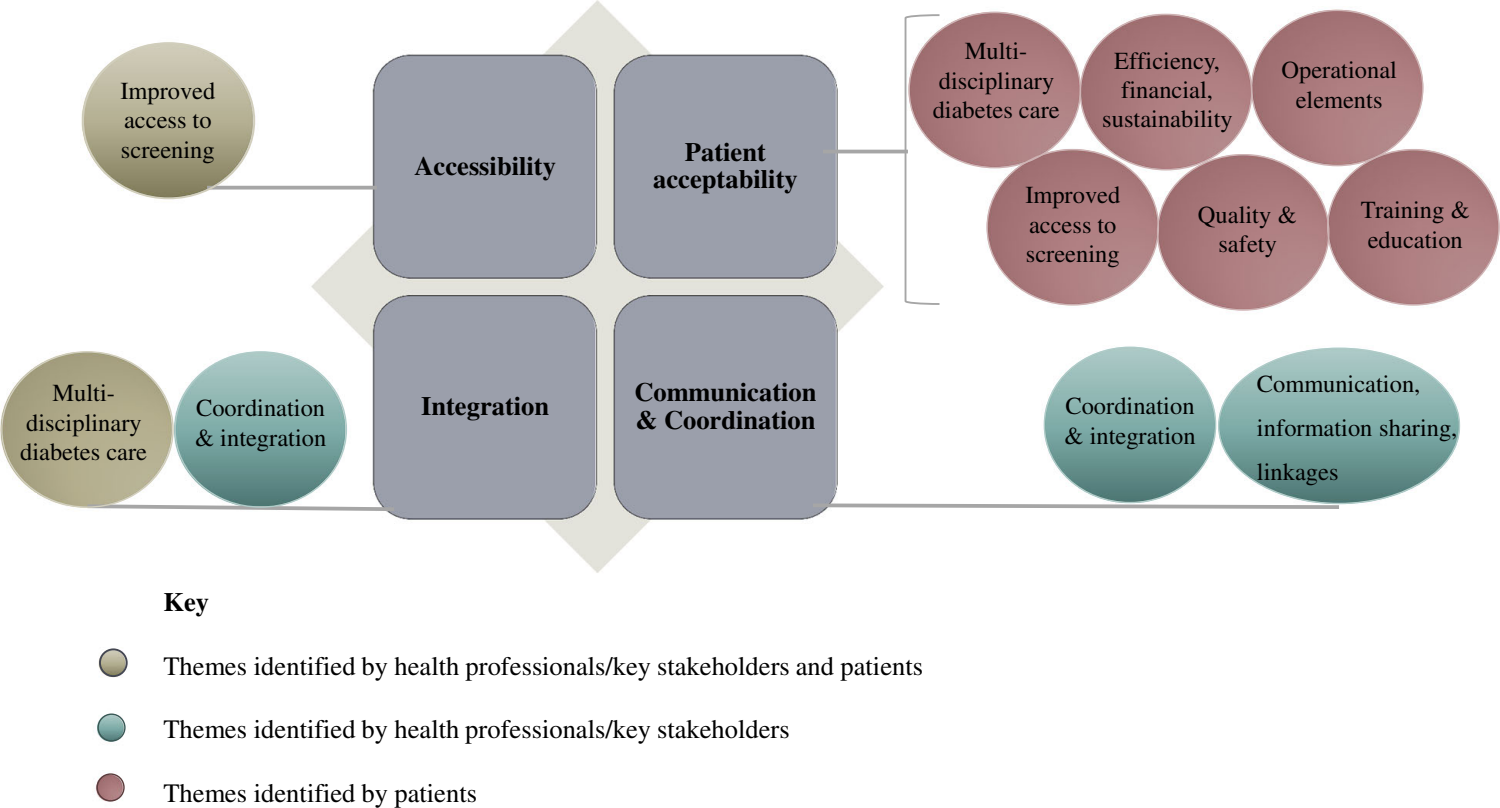
The components common to effective rural and remote DR screening models compared with themes identified by patients and health professionals. *Note: Theme 9 (suggested improvements to service delivery) is not represented*

A systematic review by the authors identified four components common to effective rural remote DR screening models, namely; accessibility, patient acceptability, integration of the service with the broader health system and communication and coordination of care [12]. Figure 5 compares those components common to effective rural remote DR screening models with themes that emerged from health professional and patient interviews. As demonstrated in a separate publication by the authors, the RODRS service has greatly improved patient access to DR screening, increasing the proportion of patients undergoing appropriate screening from 16.3% to 66.3% of patients following the implementation of the programme [11].

The RODRS service was highly acceptable to patients, with all but one patient intending to return for screening with the programme. High levels of acceptability were related to improved access to screening resulting in less travel time and periods anyway from employment, with reported positive financial consequences. Importantly, the programme met community needs, was seen as equitable and delivered a preventative health service. There is minimal international literature exploring the acceptability of rural remote DR screening models to patients. Available studies similarly report close service proximity, positive financial consequences, health personnel, camera technology and service quality as contributors to acceptability [15, 16]. However unlike this study, screening methods that allowed immediate results and feedback and culturally sensitive healthcare practices were not identified as major contributors to acceptability [15, 17]. Patients commented that greater service promotion was needed, with health professionals mentioning that methods should be tailored to the needs of the local community in terms of infrastructure, technology and health literacy. Despite poor health literacy in the region, patients had developed a basic awareness of diabetic blindness, a vital factor in delivering high re-screening rates [18–20].

The direct and timely integration of screen-positive patients with ophthalmology follow-up is common to effective rural remote DR screening models [12]. The programme sought to directly link screen-positive patients with ophthalmology follow-up, by using the same ophthalmologist to support the screening programme and provide follow-up care. However, findings suggested that many patients were not undergoing follow-up. Key barriers included poor internal communication and a lack of centralised coordination, with the programme relying on GP referral to an ophthalmologist. This has been echoed in another Australian study with GPs referring only 59% of screen-positive patients to an ophthalmologist [21]. One proposed solution was to send a list of screen-positive patients to the visiting ophthalmologists, whom then communicate back to the eye screening coordinator those patients who have received follow-up. However, this requires the commitment of an overall coordinator whose role would be to continue to monitor referrals, completed and uncompleted treatment.

Effective communication internally between health providers and externally to community organisations and health professionals was identified as a particular challenge. This was especially difficult given the vast number of providers involved in service delivery, as depicted in Fig. 4. Informal channels of communication between the eye screening coordinator, GP grader and ophthalmologist through face-to-face contact locally, were recognised as unique and particularly effective. However, more formal methods of regular communication were needed. Communication issues were exacerbated by frequent staff turnover characteristic for rural and remote areas, making established communication systems even more vital. Other rural remote DR screening models have successfully used various communication and information sharing systems such as electronic databases, communication software systems, letters, email and multi-page screening forms [22–25]. Interviewees suggested an electronic clinical database soon to be implemented in the district could promote information sharing, better identify patients with diabetes, improve tracking of screen-positive patient follow-up and potentially prevent over-servicing by recording service encounters. Better external linkages to local GPs were also identified as needed for the follow-up of non-ophthalmic issues identified by the programme. It was proposed that the screening results of all patients, regardless of whether pathology is detected, should be sent to the GP to assist in completion of the diabetes annual cycle of care.

The delivery of retinal screening with other diabetes health care produced differing opinions amongst interviewees. The multi-disciplinary service was reported to reduce patient travel, increase patient attendance and provide a comprehensive diabetes service. This is supported by the broader literature which suggests that collaborative multidisciplinary teams can improve patient outcomes and achieve higher screening rates [26–28]. However, some health professionals raised an alternative view, believing the service could be intrusive on patients, was logistically challenging and led to time inefficiencies. In contrast, patients themselves reported that the service was convenient and did not express negativity about any aspects of the multi-disciplinary service.

**Fig. 6:**
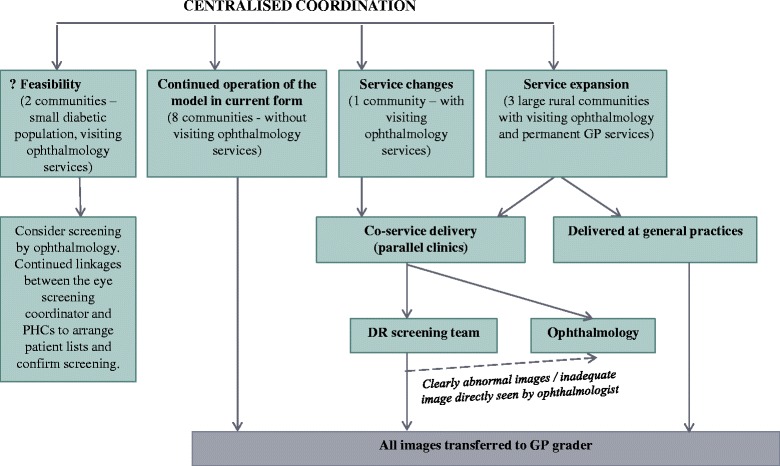
Proposed changes to the RODRS service based on interviewee feedback

Despite the challenges identified by health professionals, the RODRS programme was perceived as highly sustainable, with the potential for successful adoption or adaption in other remote communities. Figure 6 summarises potential changes to the RODRS programme and extensions of the service, based on interviewee feedback. This includes the co-location of screening and ophthalmology clinics in communities with visiting specialist services and the expansion of the service to other communities and for other pathologies. At the time of the study, two remote communities with visiting optometry and ophthalmology services had documented diabetic populations of less than six people. Thus considerations were raised in regards to the feasibility of the service in these communities and whether existing ophthalmology and optometry services should screen patients. Some health professionals suggested that in remote communities with visiting ophthalmology services, screening clinics should be delivered in a co-service capacity with ophthalmology. In addition, it was suggested that the programme be expanded to larger rural communities and operate in a co-service capacity with visiting ophthalmology services or as part of GP clinics. This was in response to patient demand for the service, to improve DR screening rates and to make more efficient use of ophthalmology services. Alternative models of DR screening could also have a role in rural centres with permanent GPs, supported by the recent use of retinal photography in GP practices with video support from specialist services [29]. The RODRS programme also has the potential to adapt with future improvements in telecommunications infrastructure in remote communities.

Other screening models have reported centralised coordination as pivotal to monitoring patient appointments and referrals at a local level and for the purposes of training, troubleshooting and quality assurance at the broader level [30–33]. The operation of multiple models of care would thus need to be delivered under a highly coordinated, centralised system designed for the entire district.

### Limitations

Interviews were conducted in three of the eleven communities visited by the programme due to difficulties accessing all eleven communities. The majority of patients interviewed were Indigenous despite the fact that Indigenous patients composed only 25% of the total patients screened by the service. This was due to a high Indigenous population in the communities where interviews were undertaken, although this group was also purposefully targeted due to lower rates of appropriate DR screening [4]. However, data saturation was reached within this sample and it is unlikely that a larger sample would have expanded the views.

## Conclusion

This innovative outreach DR screening service has shown to be highly acceptable to patients and participating health professionals. It is an accessible, equitable and holistic multi-disciplinary diabetes service that focuses on preventative health and is delivered in a way that meets the needs of the local community. It uses a limited existing health workforce efficiently in a resource poor environment. With simple changes to the programme particularly in terms of communication and coordination of care, the service could become even more successful. It is a sustainable model that can be adapted to be delivered in other remote communities nationally and potentially internationally. This could significantly increase both DR screening rates and reduce preventable blindness in rural and remote communities.

## Additional file

Additional file 1: A Semi-structured interview guides. (DOCX 104 kb)

## Abbreviations

BGL: Blood glucose level
BMI: Body mass index
DR: Diabetic retinopathy
GP: General practitioner
IHW: Indigenous health worker
NPDR: Non-proliferative diabetic retinopathy
PDR: Proliferative diabetic retinopathy
PHC: Primary health care centre
PTS: Patient travel subsidy scheme
RODRS: Remote Outreach DR Screening

## Footnotes

1 Indigenous refers to Aboriginal peoples and/or Torres Strait Islanders.

2 Note: the term health professional or health practitioner incorporates themes identified in both health professional and key stakeholder interviews.

3 The ‘MAP of medicine’ IT system is a system of locally customised pathways, centrally controlled referral forms and clinical information [14].
