# Supplementary material for: What do patients with diabetes and providers think of an innovative Australian model of remote diabetic retinopathy screening? A qualitative study
Source: BMC Health Serv Res. 2017 Feb 22;17:158. doi: 10.1186/s12913-017-2045-2 (PMC5320669; doi:10.1186/s12913-017-2045-2)
Supplement: Additional file 1: — A Semi-structured interview guides. (DOCX 104 kb) [file 12913_2017_2045_MOESM1_ESM.docx]

**Additional file 1**

***Semi-structured interviews with nurse screeners and the eye screening coordinator participating in the RODRS programme***

1. Can you describe your role in the RODRS service? How long have you been involved?
2. How did this programme develop? Why was it introduced?

What eye screening services were available prior to the introduction of the service and where were they operating?

1. Can you describe how the screening programme is organised and operates?

- How are diabetic patients identified and invited for screening? How are appointments made?
- Can you describe the screening process?
- What health professionals are involved in the programme and what are their roles?
- How are patients notified of positive screening results? How is follow-up organised? How could this be improved (if at all)? Do any screen-positive patients fail to attend?

1. Do you believe the implementation of this service has affected patient’s willingness and ability to access screening?

Do you believe patient recruitment to the programme could be improved? If so, how?

1. How would you describe communication and interactions between the GP graders, ophthalmologists and the screening team? Do you believe this could be improved?
2. What are your thoughts generally about the programme? Do you think it has been successful or unsuccessful? Why?
3. What do you recognise as the benefits of the programme?
4. What do you recognise as the drawbacks of the programme? Are there any areas of the model’s operation that you feel could be improved?

Has the programme changed or been improved over its two years of operation? What lessons have been learnt?

Do you believe that it is sustainable? Do you believe it could be successfully trialled in other remote communities?

1. Are there any other means by which patients are getting their eyes screened apart from through the RODRS programme? If yes, why do you believe they are accessing those screening services?
2. Is there anything else you would like to add that I have not asked you?

**Thank you very much for your time today**

***Semi-structured interviews with GP graders participating in the RODRS programme***

1. Can you describe your role in the RODRS programme? How long have you been involved?
2. How did this programme develop? Why was it introduced?

What eye screening services were available prior to the introduction of the service and where were they operating?

1. What is your understanding of the RODRS programme? Can you describe how it operates once the screening team has captured patient images?
2. How successfully do you believe the programme links screen-positive patients with specialist follow-up?
3. How would you describe communication and interactions between the GP graders, ophthalmologists and the screening team? Do you believe this could be improved?
4. What were your experiences completing the training required to become a GP grader? Do you believe this could this be improved?
5. What are your thoughts generally about the programme? To what degree do you think it has been successful or unsuccessful? Why?
6. What do you recognise as the benefits of the programme?
7. What do you recognise as the drawbacks of the programme? Are there any areas of the programme’s operation that you feel could be improved? Do you believe that it is sustainable? Do you believe that it could be successfully trialled in other remote communities?
8. Are there any other means by which patients are getting their eyes screened apart from through the RODRS programme? If yes, why do you believe they are accessing those screening services?
9. How does the model impact on your clinical practice?
10. Is there anything else you would like to add that I’ve not asked you?

**Thank you very much for your time today.**

***Semi-structured interviews with ophthalmologists participating in the RODRS programme***

1. Can you describe your role in the RODRS programme? How long have you been involved?
2. How did this programme develop? Why was it introduced?

What eye screening services were available prior to the introduction of the service and where were they operating?

1. What is your understanding of the RODRS programme and how it operates?
2. How successfully do you believe the model links screen-positive patients with specialist follow-up?

How efficient do you believe this system is? Why? Have any challenges been encountered integrating screening with treatment?

1. How would you describe communication and interactions between the GP graders, ophthalmologist and the screening team? Do you think this could be improved?
2. What are your thoughts generally about the programme? To what degree do you think it has been successful or unsuccessful? Why?
3. What do you recognise as the benefits of the programme?
4. What do you recognise as the drawbacks of the programme? Are there any areas of the programme’s operation that you feel could be improved?

Do you believe that this model is sustainable? Do you believe it could be successfully trialled in other remote communities?

1. Has the implementation of the programme impacted on the type and/or volume of clinical work undertaken during your visits to the region? How do you feel the introduction of the programme has impacted on your clinical practice?
2. Is there anything else you would like to add that I’ve not asked you?

**Thank you very much for your time today.**

***Semi-structured interviews with the diabetes educator / Indigenous outreach worker participating in the RODRS programme***

1. Can you describe your role in the RODRS service? How long have you been involved?
2. Can you describe how the screening programme is organised and operates?

- How are diabetic patients identified and invited for screening? How are appointments made?
- Can you describe the screening process?
- What health professionals are involved in the programme and what are their roles?
- How are patients notified of positive screening results? How is follow-up organised? How could this be improved (if at all)? Do any screen-positive patients fail to attend?

1. Do you believe the implementation of this service has affected patient’s willingness and ability to access screening?

Do you believe patient recruitment to the programme could be improved? If so, how?

1. What are your thoughts generally about the programme? Do you think it has been successful or unsuccessful? Why?
2. What do you recognise as the benefits of the programme?
3. What do you recognise as the drawbacks of the programme? Are there any areas of the model’s operation that you feel could be improved?

Has the programme changed or been improved over its two years of operation? What lessons have been learnt?

Do you believe that it is sustainable? Do you believe it could be successfully trialled in other remote communities?

1. Are there any other means by which patients are getting their eyes screened apart from through the RODRS programme? If yes, why do you believe they are accessing those screening services?
2. Is there anything else you would like to add that I have not asked you?

**Thank you very much for your time today.**

***Semi-structured interviews with optometrists in regards to the RODRS programme***

1. Could I ask you to give a brief overview of your understanding of the RODRS programme and how it operates?
2. What is your opinion of the impact of the RODRS programme?
3. Can you describe how diabetic eye screening differed prior to the implementation of the model? Why do you think this programme was introduced?
4. To what degree do you think the RODRS programme has been successful or unsuccessful? Why?
5. What do you recognise as the benefits of the programme?
6. What do you recognise as the drawbacks of the programme? Are there any areas of the programme’s operation which you feel could be improved in the future?
7. How do you believe the programme has impacted on the local workforce?

Do you believe it is an efficient or inefficient model of health care delivery and why?

Do you believe that it is sustainable? Do you believe it could be successfully trialled in other remote communities?

1. Are there any other means by which patients are getting their eyes screened apart from through the RODRS programme?
2. Is there anything else you would like to add that I’ve not asked you?

**Thank you very much for your time today.**

***Semi-structured interview with the manager of the health district in regards to the RODRS programme***

1. Could I ask you to give a brief overview of your understanding of the RODRS programme and how it operates?
2. What is your opinion of the impact of the RODRS programme?
3. Can you describe how diabetic eye screening differed prior to the introduction of the model? Why do you think this programme was introduced?
4. To what degree do you think the screening model has been successful or unsuccessful? Why?
5. What do you recognise as the benefits of the programme?
6. What do you recognise as the drawbacks of the programme? Are there any areas of the model’s operation which you feel could be improved in the future?
7. Can you comment on how successful/unsuccessful communication and coordination of the programme has been?
8. How do you believe the programme has impacted on the local workforce?

Do you believe it is an efficient or inefficient model of health care delivery and why?

Do you believe that it is sustainable? Do you believe it could be successfully trialled in other remote communities?

1. Can you please comment on the attached diagram, ‘*proposed changes to the delivery of the RODRS programme’*?
2. Is there anything else you would like to add that I’ve not asked you?

**Thank you very much for your time today.**

***Semi-structured interviews with patients participating in the RODRS programme***

**DOB**:

**Gender**: MALE FEMALE

**Previous DR screening:** YES NO Details

1. How long have you had diabetes (sugar problems)? How do you think diabetes (sugar problems) might affect your eyes?
2. Have you come to this eye check clinic before?

- If yes, when and did they find any problems with your eyes?

Before the eye check clinic came, did you get your eyes checked?

- If yes, can you tell me about that? [Where/ with whom / how often]. What was good and not so good about those eye checks?

1. I would like to ask you some questions about coming to this eye clinic:

- How did you find out that they do eye checks here?
- What happens when you come to get your eye checks here?
- How do you know if your eyes are good or bad after your eye check?
- Do you know when you will next need an eye check?

1. What do you think about this eye check clinic? Would you come back to have your eyes checked here? Why?
2. What are the benefits (the good things) for you in being able to get your eyes checked here?
3. Do you think anything else could be done to make getting your eyes checked here better? If so what?
4. Is there anything else you would like to add that I’ve not asked you?

Do you identify as Aboriginal and/or Torres Strait Islander?

**Thank you very much for your time today**
